# Bodily Illusions in Young Children: Developmental Change in Visual and Proprioceptive Contributions to Perceived Hand Position

**DOI:** 10.1371/journal.pone.0051887

**Published:** 2013-01-30

**Authors:** Andrew J. Bremner, Elisabeth L. Hill, Michelle Pratt, Silvia Rigato, Charles Spence

**Affiliations:** 1 Sensorimotor Development Research Unit, Department of Psychology, Goldsmiths, University of London, London, United Kingdom; 2 Department of Experimental Psychology, Oxford University, Oxford, United Kingdom; University of Reading, United Kingdom

## Abstract

We examined the visual capture of perceived hand position in forty-five 5- to 7-year-olds and in fifteen young adults, using a mirror illusion task. In this task, participants see their left hand on both the left and right (by virtue of a mirror placed at the midline facing the left arm, and obscuring the right). The accuracy of participants’ reaching was measured when proprioceptive and visual cues to the location of the right arm were put into conflict (by placing the arms at different distances from the mirror), and also when only proprioceptive information was available (i.e., when the mirror was covered). Children in all age-groups (and adults) made reaching errors in the mirror condition in accordance with the visually-specified illusory starting position of their hand indicating a visual capture of perceived hand position. Data analysis indicated that visual capture increased substantially up until 6 years of age. These findings are interpreted with respect to the development of the visual guidance of action in early childhood.

## Introduction

Accurately representing the disposition of our body and limbs in space is vital if we are to manipulate and move around our environments in a competent manner. To form such body representations, we need to integrate the spatial information about the limbs arriving from multiple sensory modalities (vision, proprioception, touch, and audition) [1]–[3]. Even though young infants perceive commonalities of information across these sensory modalities (e.g., [4]–[7]), it is likely that the neural mechanisms underlying representations of the body and the peripersonal space that surrounds it undergo significant postnatal development; any early ability to represent the layout of the body would need to be retuned throughout development in order to cope with physical changes in the disposition, sizes, and movements of the limbs which continue even beyond adolescence (see [8]).

The provision of multiple sources (modalities) of sensory information about the body bestows functional advantages as they provide complementary information about it and also permit greater confidence in sensory estimation than does one modality alone [9], [10]. As adults, we integrate these multiple signals into unified representations. But the ease with which we accomplish this feat belies its computational complexity. For not only do the senses convey information about the environment in different spatial codes (e.g., somatosensory stimuli are initially coded with respect to the body surface, whereas visual stimuli are initially coded in a retinocentric frame of reference), but the relationship between the senses changes whenever we change posture (e.g., when the eyes move in their sockets [11]), or when the body changes shape as children grow [8], [12].

One of the ways in which adults approach the problem of integrating the senses is to weight information from the most reliable modality most heavily. When localizing a limb (e.g., a hand), greater weighting of the visually-derived location of the hand, as compared to the proprioceptive location, will normally lead to more accurate localization because of the greater reliability of visual spatial information. This tendency to rely on vision of the limbs can be observed in bodily illusions such as the “rubber hand illusion” [13] and the “mirror illusion” [14], in which visual information specifying the presence of a hand, biases a person’s estimate of where their own hand is located.

Although no studies that we know of have directly examined the development of visual capture in spatial localization of the limbs outside of the on-line guidance of actions, a number of researchers have asserted that vision generally becomes more dominant in manual spatial localization over the course of childhood [15], [16]. Although we now know that this does not occur in all aspects of sensorimotor development (see, e.g., [17]), support for this assertion in at least one context has been provided quite recently by [18]. On the basis of findings from a tactile localization task, these researchers demonstrated that children develop in the extent to which they rely on a visual external frame of reference for locating tactile stimuli.

While this research suggests that spatial representations of the body and limbs may become increasingly visual in nature across early development, this need not necessarily be the case. Firstly, it is important to note that a reliance on vision even in adults is not the rule when locating the limbs. As demonstrated in a number of different multisensory situations, adults weight the senses in proportion to their reliabilities within the context of the current task, thus maximizing the reliability of the combined estimate [9]. Under this framework, the “dominant” modality is not relied upon exclusively; it is just weighted to a greater extent than other modalities in proportion to its relative reliability. Thus, researchers have shown that when vision is no longer the most reliable sense, other modalities such as proprioception are given a greater weighting (e.g., [19], [20]). Under such an approach, there is no reason to assume that development would inevitably converge on greater visual weighting in perceived limb position. Indeed, given developmental changes in the acuity of the senses contributing to perceived limb position (e.g., [21], [22]) it is quite possible that the optimal weightings of the senses would continue to change right across childhood.

Secondly, as mentioned above, visual weighting actually declines in some sensorimotor tasks across development (e.g., balance; see [23]). Furthermore, it does not inevitably follow that multisensory spatial representations of the body undergo the same developments as spatial representations of external objects or stimuli impinging on the body (the tactile stimuli used in Pagel et al.’s study [18] can be considered as extrapersonal in the sense that they derive from objects apart from the body). A number of authors have suggested that adults may perceive bodily stimuli with respect to different spatial frames of reference (internal and/or external) depending on the task at hand [24], [25], and it is quite plausible that such internal and external frames of reference emerge according to different developmental time-courses [8].

In this paper, we report the findings of an experiment in which we investigated the occurrence of visual capture of limb position during early childhood. We utilised Holmes et al.’s [14] “mirror illusion” task as a means of comparing the extent of visual capture of limb position as measured by post-illusion reaching behaviours in 5- to 7-year-old children.

## Methods

### Design

**Figure 1:**
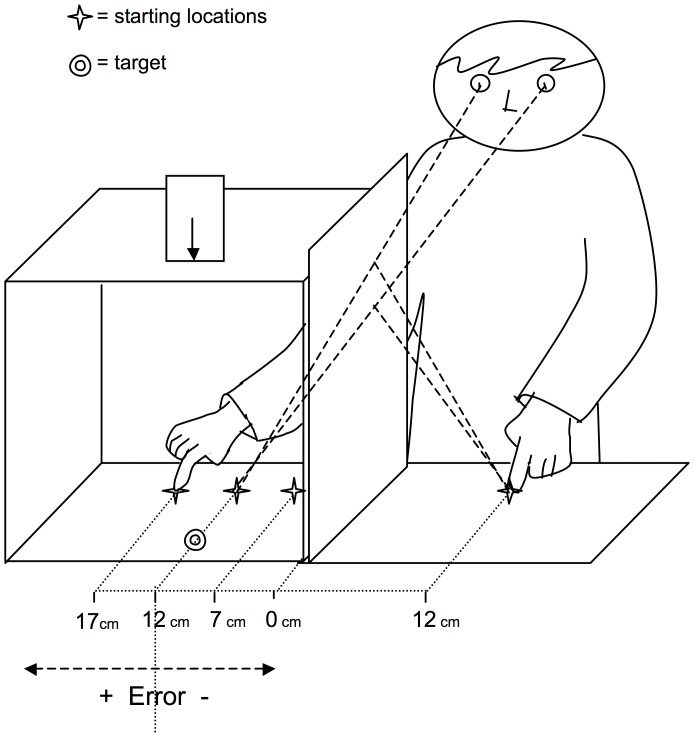
Mirror apparatus from the experimenter’s point of view. The scale below the diagram indicates distance from the mirror towards the participant’s right. Participants viewed their left hand on both the left and right of their midline (by virtue of a mirror placed at the midline facing the left arm, and obscuring the right arm). The left hand was placed 12****cm from the mirror. The position of the participant’s hidden right hand was either congruent with the visual image (12****cm to the participant’s right with respect to the mirror), or else was put into spatial conflict in the azimuthal dimension with the illusory visual image (7****cm or 17****cm to the participant’s right with respect to the mirror). The location of the left hand, and all of the starting locations of the right hand were 23****cm in front of the participant’s body. Participants reached toward the target (12****cm to the right of the mirror, and 48****cm in front of the participant’s body – indicated by the visible arrow above it). Lateral terminal errors were measured. Errors to the participant’s right (left) with respect to the target were scored as positive (negative).

We presented children with the Mirror Illusion [14], in which they viewed one of their hands on both the left and right of their midline (via a mirror placed at the midline facing one arm and obscuring the other; see Fig. 1). In this illusion, when the hidden right hand (perceived proprioceptively) is put into spatial conflict with the illusory visual image, adult participants’ perception of the location of their hidden hand and also their subsequent reaches with that hand are typically biased (partially captured) by the visual illusory information about the initial position of their hand [14], [26], [27]. We measured the extent of visual capture in our developmental sample by asking children to reach to a visible target with the hand on which the mirror illusion had been induced (the hidden hand; see Fig. 1) and examining the extent to which their reaches were biased by illusory visual cues concerning the position of the hand before the reach was executed.

We measured children’s lateral reaching errors to the target location by measuring the distance, in the axis extending perpendicularly from the mirror surface, between the point where their index finger landed, and the target location. Errors away from the target location and toward the mirror were scored as negative, and those away from the mirror and target were scored as positive (see Fig. 1). The participant’s left (non-reaching) hand was placed 12****cm to the left of the mirror throughout the experiment, yielding a (illusory) mirror image of a hand seen 12****cm to the right of the mirror. We compared reaching errors with the hidden hand across three different starting locations: 7****cm, 12****cm, and 17****cm to the right of the mirror. Thus the mirror image only gave veridical visual information about the location of the reaching hand when it was placed 12****cm to the right of the mirror. The mirror illusion, if it occurred, was thus predicted to give rise to negative reaching errors when the participant’s hand was placed at a starting position of 7****cm, and positive errors when placed at 17****cm. In addition to the starting position variable, the availability of visual information concerning the location of the hand was also manipulated by either covering the mirror, or else leaving it uncovered.

Mirror and No mirror trials were conducted in two separate blocks, the order of which was counterbalanced across participants. Within each block the participant received 6 trials at each of the starting positions; thus, 18 trials per block, and 36 trials in total, presented in a random order.

### Participants

Forty-five children aged between 4 and 7 years took part in this study. We divided the children into 3 age-groups centred around the mean ages of 5 years (54–65 months), 6 years (66–77 months), and 7 years (78–89 months) (see Table 1). To confirm replication of Holmes et al.’s [14] paradigm, we also tested 15 adults (see Table 1). Data were included from all participants apart from one 5-year-old boy, who failed to complete the task. All children were tested in their primary school, and all adults were tested in the university.

**Table 1:** Participant characteristics.

Age-group	n	Gender split	Mean age in months or years	SD of age in months or years
54–65 months	14	11 m, 3 f	61.7 months	2.5 months
66–77 months	16	8 m, 8 f	72.7 months	3.8 months
78–89 months	15	8 m, 7 f	83.4 months	3.2 months
Adults (18–35 years)	15	7 m, 8 f	26.2 years	5.0 years

### Ethics Statement

Ethical approval was gained from the Research Ethics Committee at Goldsmiths, University of London prior to testing. For child participants, written informed consent was obtained from parents or guardians prior to testing. Verbal assent was also obtained from the children themselves. For adult participants, written informed consent was obtained prior to testing.

### Apparatus

Figure 1 presents a schematic diagram of the experimental apparatus. A 45×30****cm mirror was mounted on a table with its reflective surface facing the participant’s left side. On the table to the participant’s left, a mark indicated the location where the experimenter instructed participants to place their left index finger during the course of the experiment. To the right of the mirror a raised platform, with a curtain attached to drape over the shoulder, obscured the participant’s right arm from view. A picture of “Lady” from “Lady and the Tramp” was placed on top of the platform, with a target arrow pointing downward. This arrow functioned as the target indicator. Participants pointed directly below this indicator on the surface of the table. Underneath the platform there were three marks, visible only to the experimenter. These marks indicated to the experimenter where to place the participants’ finger before asking them to reach toward the target.

### Procedure

Children were introduced to the mirror box apparatus by placing their hands 12****cm each side of the mirror (in this layout, vision and proprioception are not in conflict) and asking them to tap their index fingers synchronously whilst inspecting the mirror image (see [14]). The experimenter asked the participant if the mirror-image hand looked like their own right hand. Once the participant answered “yes”, there then followed a series of practice trials. In these trials, the experimenter placed the index finger of the participant’s right-hand 12****cm from the mirror, and asked him/her to visually inspect the mirror image of this hand whilst tapping both index fingers synchronously on the surface of the table. Once it had been confirmed that visual inspection and synchronous finger-tapping had occurred, the participant was instructed to look at the target arrow and reach with the hidden hand to touch a location directly below it on the table surface. Once the participant had achieved three reaches which fell within a 2****cm×2****cm square surrounding the target point the first experimental trial began. Mirror trials were exactly the same as the practice trials, except that the participant’s hidden right-hand index finger was placed on one of the three starting locations by the experimenter. No Mirror trials were identical to Mirror trials except that the experimenter gave no directions regarding where to look whilst synchronously tapping the fingers.

### Statistical Analyses

All analyses were conducted using IBM SPSS Statistics Version 19. In the reported Analysis of Variance (ANOVA), Greenhouse-Geisser corrections are applied to all p values where necessary. Preliminary analyses revealed no significant main effects of gender, or interactions of gender with other factors, and so the reported analyses do not include gender as a factor.

## Results

**Figure 2:**
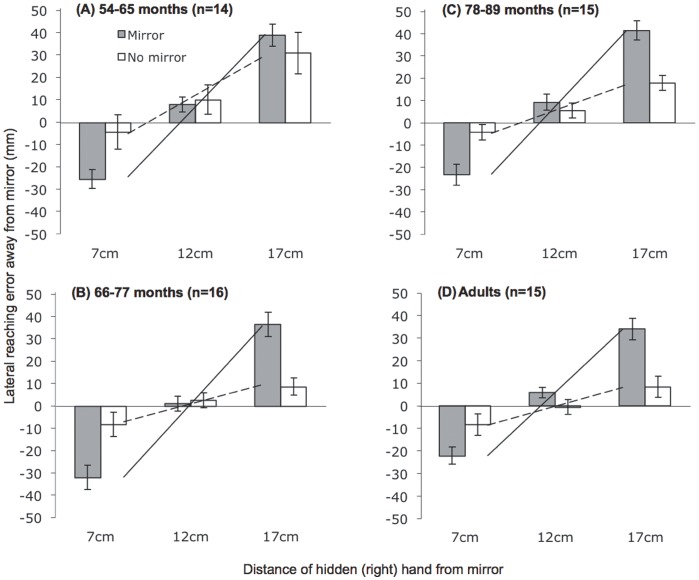
Mean reaching errors for each age-group across starting positions and mirror conditions. Bars represent the errors within each condition (shaded bars = Mirror, unshaded bars = No mirror). The superimposed lines represent plots of the gradient of mean reach errors against starting position (solid line = Mirror, dashed line = No mirror).

Figure 2 presents separate plots of the reaching errors made by each age-group of children and also the adult group. In each plot, reaching errors at each of the separate starting locations are compared across conditions in which visual information in the mirror concerning the location of the participants’ right hand was varied (“Mirror” and “No Mirror” conditions). The participants’ use of illusory (non-veridical) visual information is indicated by reaching errors under conditions of crossmodal conflict (i.e., when non-veridical visual and veridical proprioceptive information about the hand conflict). In the “Mirror” condition, a reliance on visual information predicts negative errors from a starting position of 7****cm, errors around zero from a starting position of 12****cm, and positive errors from a starting position of 17****cm.

**Figure 3:**
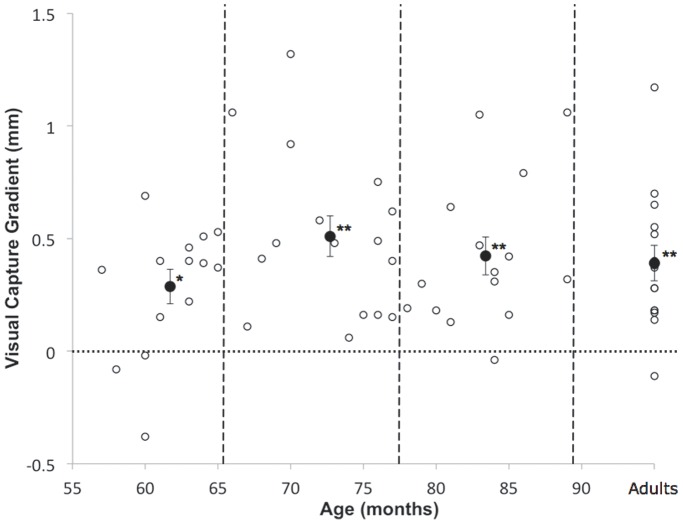
VCG scores plotted for each participant against age in months. The visual capture score presented here is a difference of gradients. It is calculated by comparing the gradients of reach error (mm) against starting position (mm) in the Mirror and No Mirror conditions (error gradient in the “Mirror” condition - error gradient in the “No Mirror” condition). Open circles indicate individual participants’ visual capture gradient scores. Vertical dashed lines separate the age-groups compared in the analysis. Closed circles with standard error bars indicate the mean VCG scores for each age-group. Asterisks indicate group means which are reliably greater than chance (0) (* = p≤.01, ** = p.≤001).

To construct a measure of visual capture, we calculated best-fit regression lines of reaching errors (mm) against starting location (mm), and derived gradients for these regression lines in the “Mirror” and “No mirror” conditions (see plotted lines in Fig. 2). In the “Mirror” condition, error gradient magnitudes yield a measure of the extent of error across visual conflict conditions (i.e., independent of direction), which can be set against a baseline error gradient calculated from the “No Mirror” condition. Because these gradient difference scores, which we refer to as Visual Capture Gradient (VCG) scores, measure the error induced by the mirror as a function of the degree of crossmodal conflict (rather than the absolute location of the hand), they have the advantage of providing an index of reliance on the illusory visual information which is independent of other factors which may vary across the mirror condition such as postural change or proprioceptive drift [28], [29]. Figure 3 plots VCG scores for each participant against their age in months. All age-groups demonstrated a VCG score that was significantly greater than zero (chance), 54–65 months: t(13) = 3.76, one-tailed p<.01, d = 1.01; 66–77 months: t(15) = 5.64, p<.001, d = 1.41; 78–89 months: t(14) = 4.99, one-tailed p<.001, d = 1.29; Adults: t(14) = 4.99, one-tailed p<.001, d = 1.29).

To examine whether there were any developmental changes in the visual capture of hand position across early childhood we compared the VCG score across the three age-groups of children shown in Figures 2 and 3. A one-way ANOVA (Age-group: 54–65/66–77/78–89 months), revealed no main effect of age-group (F(2,42) = 1.73, n.s., η_p_^2^ = .076). Nonetheless, closer inspection of the changes in VCG in Figure 3 indicates that developmental increases may be taking place at a more fine-grained level within the youngest of the age-groups whom we tested. As recent findings from Pagel and colleagues [18] indicate that children’s use of an external (likely visual) frame of reference for locating tactile stimuli emerges up until the 6^th^ birthday, we explored age-related changes in VCG scores within all of the children under 6 years of age. A post-hoc correlation of VCG score against age in months revealed a substantial and significant increase in visual capture within this group, r(20) = .61, p<.01.

## Discussion

The study presented here demonstrates that young children are susceptible to the visually-induced mirror illusion in which the perceived location of a hand hidden from view is biased by the mirror image of the participant’s other hand. These findings converge with recent evidence that children between the ages of 5 and 9 years of age are susceptible to the Rubber Hand Illusion in which a hidden hand is biased by a visually presented fake hand [30]. The visual capture of reaching shown by all of the age-groups we tested demonstrates that, even from 5 years, children like adults use vision in addition to proprioception when locating their hands in the azimuthal plane [14], [20], [30].

The observation of a visual capture of hand position in all of the age-groups we tested raises the question of whether even younger children and also infants rely on visual cues to hand position. Certainly, it seems likely that, from early in life, infants can register the necessary multisensory correspondences between vision and signals arising from the limbs. It is now well established that infants as young as 3 months of age perceive spatiotemporal correspondences between the felt movements of their own limbs and an on-screen image of that movement [4], [6], [7], [31] (see [32] for a review). Furthermore, Bremner and colleagues [33] have argued that a visual spatial code influences infants’ responses to tactile stimuli at 6.5 months of age. The existence of such early multisensory abilities, and similar skills observed even in newborns [34]–[36] indicates that vision may play a role in hand position throughout early development.

However, despite finding visual capture of reaching in all age-groups, exploratory analyses of our data also indicated significant developmental increases in the weighting given to vision between 57 and 72 months (4¾ years and 6 years). The finding that the visual capture of hand position increases with age is in keeping with the general claims made by Renshaw [15] and Warren and Pick [16], and, more recently, by Pagel et al. [18], that children become increasingly reliant upon vision in their reaching and other orienting responses to external targets over early childhood. In the study reported here, the reaches that children made towards a target were increasingly biased by visual cues to the hand given prior to the onset of the reach (children received no visual feedback during their reaches). Thus, the results reported here demonstrate that the developmental trend towards greater visual weighting when orienting towards targets is also apparent in children’s developing representations of their limbs.

But what developing processes might underlie the increase in the visual influence on hand position in early childhood? One possibility is that the emergence of visual weighting could be explained by a progressive linking of visual and proprioceptive modalities in early childhood. Warren and Pick [16], following on from Birch and Lefford [37], posited just such an idea, suggesting that increased visual reliance across childhood is made possible by a progressive general linking of the senses. However, this account has difficultly explaining evidence, described above, demonstrating the ability to register multisensory correspondences in much younger infants.

Our preferred interpretation is that the emergence of the visual capture of hand position documented here, occurs as part of a developmental process of multisensory fine-tuning (rather than a registering of multisensory correspondence per se), in which the specific weightings of the senses are modified in order to improve the efficiency of sensorimotor performance. In fact, the literature on visually-guided reaching gives us some clues as to why the visual capture of perceived hand position might increase in early childhood. A number of studies investigating children’s reliance on sight of the hand when reaching towards an external target indicate developmental increases in the use of visual feedback when reaching.

For instance, Smyth, Peacock, and Katamba [38] found that, between 7 to 9 years, children increase in the extent to which they gain speed advantages from vision of the hand. Others have observed non-monotonic shifts in the influence of visual feedback on reaching. Hay [39] examined what percentage of children’s reach trajectories would be influenced by sight of the hand moving in an incorrect trajectory towards the target (caused by their wearing prismatic lenses). She showed that whereas 5-year-olds’ reaches towards the targets were relatively unaffected by this conflicting visual feedback (only 6% of their reach trajectory), this visual influence increased sharply (to about 13%) by 7 years of age, before levelling off to 8–9% by 9–11 years.

Thus, visual influence on perceived hand position, both in a stationary context (as in the current study) and in the more dynamic context of guided reaching tasks, appears to increase during early to middle childhood. An important task for future research will be to determine the extent to which developmental tuning of multisensory representations of the body occur in a generalised way or are specific to particular sensorimotor tasks as they are in adults [20].
